# Type D personality, social support and depression in patients with diabetes: a structural equation model

**DOI:** 10.3389/fendo.2026.1652252

**Published:** 2026-02-03

**Authors:** Yingjie Lin, Na Xu, Yi Sui, Qiangwei Zou, Jinglan Zhang, Li Zhao

**Affiliations:** 1 School of Geriatrics and Elderly Care Industry, Shenyang Medical College, Shenyang, Liaoning, China; 2 Department of Nursing, Beijing Health Vocational College, Beijing, China; 3 Department of Neurology, The Fourth People’s Hospital of Shenyang, China Medical University, Shenyang, Liaoning, China; 4 Shenyang Red Cross Hospital, Shenyang, Liaoning, China

**Keywords:** depression, Diabetes, social support, structural equation modeling, type D personality

## Abstract

**Objective:**

To investigate the current status of depression among patients with diabetes and the influencing factors, and to analyze the relationship between Type D personality, social support and depression.

**Methods:**

This study is a multi-center cross-sectional study. Using the cluster random sampling method, 1000 patients with diabetes were randomly selected from 6 tertiary hospitals in Shenyang from June to August 2024. Data was collected using a questionnaire consisting of the Type D Personality Scale, Perceived Social Support Scale, and Self-rating Depression Scale. Statistical analysis was conducted using SPSS 22.0 and AMOS 24.0. χ^2^ test, logistic regression model, and decision tree model were employed to investigate the influencing factors of depression in patients with diabetes. The predictive performance of the logistic regression and decision tree models was compared using the ROC curve. Pearson correlation analysis was conducted to examine the correlations between key variables. Additionally, a structural equation model was constructed to explore the relationships among type D personality, social support, and depression, as well as to evaluate the mediating role of social support.

**Results:**

The ROC curve analysis indicated that the predictive performance of the logistic regression model was marginally superior to that of the decision tree model. Structural equation modeling demonstrated a significant negative association between social support and type D personality (*β* = -0.669, *P* < 0.01) as well as between social support and depression (*β* = -0.370, *P* < 0.01). When social support was treated as a mediating variable, the absolute value of the path coefficient between type D personality and depression decreased (*β* = 0.628, *P* < 0.01), suggesting a partial mediating effect.

**Conclusion:**

The prevalence of depression is higher in patients with diabetes, and type D personality in patients with diabetes increases their risk of developing depression, while social support plays a crucial role in maintaining and regulating mental health, which may reduce the risk of depression. Patients with diabetes who have a type D personality tendency may mediate the development of depression through their perception of social support. Therefore, interventions should aim to strengthen social support for these patients, improve their ability to utilize it, mitigate type D personality traits, and ultimately enhance their mental health.

## 1 Introduction

Currently, diabetes is considered to be one of the most common chronic diseases in the world. With the development of modern society, living standards have improved, and eating habits are also quite different from the traditional ones. Modern people prefer high-oil, high-salt and high-sugar diets, leading to an increase in the prevalence of obesity. At the same time, the incidence rate of diabetes is also increasing year by year. According to the 10th diabetes map in 2021, the total number of patients with diabetes in the world is about 537 million, which is predicted to increase to 643 million by 2030 and 783 million by 2045. Among the existing patients with diabetes, the number of Chinese patients has reached 114 million. In China, the prevalence of diabetes is 12.8%, indicating that nearly 13 people in every 100 people have diabetes (1). Other studies have shown that (2, 3), patients with diabetes often suffer from psychological and mental diseases, of which depression and anxiety are the main diseases. Diabetes has become a prominent global public health problem and an important cause of death, disease burden, and economic burden.

Depression, as a serious mood disorder, can lead to significant deterioration of physical and social functions (4). More importantly, diabetes has a long course of the disease, which easily causes more complications, resulting in a heavy mental burden for patients, depression, anxiety, and other negative emotions, thus affecting treatment compliance, leading to poor blood glucose control, and a decline in patients’ living standards. As the Chinese mental health survey recorded in the Blue Book of Depression shows, the incidence rate of depression among Chinese adult population is 3.4%. Many studies have shown that depression can affect the occurrence and development of diabetes by influencing patients’ health-related behavior (5, 6). The question of whether depression is a risk factor for diabetes remains controversial. However, studies indicate that the incidence of depression in patients with diabetes is approximately two to three times higher than in those without diabetes. Studies have found that patients with diabetes have a high burden of depression, with an incidence rate of about 31% (7) and a reported prevalence in China of 25.9% (8). In addition, depression generates negative emotions that reduce self-management, treatment compliance, and the abilities for interpersonal relationship management and self-care in patients with diabetes, ultimately contributing to a worsening of their disease status.

Personality attributes are closely related to the occurrence and development of diseases, and indirectly or directly affect individual mental health. Among them, type D personality is considered to be a risk factor for depression in patients with diabetes (8). Type D personality, also known as “depressive personality”, is characterized by negative emotions and social suppression. Individuals with type D personalities are more likely to perceive negative emotions such as loneliness, sadness, and anger in daily life and suppress the expression of emotions (7, 9). Some studies have shown that type D personality is significantly related to diabetes related complications. Moreover, the ability of patients with diabetes with type D personality to comprehend social support is lower than that of patients with non-type D personality, and type D personality is one of the influencing factors of social support of patients.

Social support refers to the personal care and help from others from the social network, including objective material support and subjective emotional support. The types of social support mainly include spiritual and material help and support given to individuals by family members, relatives, friends, community organizations (10). Social support plays a buffer role in mental health, helping to avoid psychological barriers, improve patients’ self-management level, improve lifestyle, and enhance blood glucose control (11, 12). Research indicates that low social support is associated with depression, reduced treatment compliance, and poor glycemic control in patients with diabetes (13–15), all of which contribute to a decline in their physical and mental health. In addition, family-related social support is significant in improving the treatment compliance of patients with diabetes (16). The less social support an individual feels, the more likely it is to have negative emotions such as anxiety and depression, to improve the ability to feel social support and reduce the occurrence of negative emotions.

Many studies have explored the relationship between social support and depression. These studies focus on understanding how social support affects depression through mediating factors, or on the mediating role of social support as a mediating variable between type D personality and depression in the elderly. However, in patients with diabetes, limited studies have not clarified the relationship between the three. Some studies have provided relevant evidence that there is a negative correlation between social support and depression in elderly patients with confirmed diabetes. Type D personality has a positive predictive effect on depression in patients with diabetes foot ulcers. Patients with diabetes who have a type D personality tend to have lower levels of social support. Based on this, according to the social support theory, individuals’ psychological or material support in social relations can alleviate the negative effects of other factors on physical and mental health. We put forward the following four hypotheses.

Hypothesis 1: Type D personality was positively correlated with depression.
Hypothesis 2: Type D personality was negatively correlated with social support.
Hypothesis 3: Social support was negatively correlated with depression.
Hypothesis 4: Social support played a mediating role between Type D personality and depression.

## 2 Materials and methods

### 2.1 Research participants

In this study, A cross-sectional study was employed to select patients with diabetes who were hospitalized in six tertiary hospitals in Shenyang City from June to August 2024 to conduct a questionnaire survey. The inclusion criteria were as follows: (1) Age ≥18 years old; (2) Meeting the clinical diagnostic criteria for diabetes, fasting plasma glucose greater than or equal to 7.0 mmol/L or plasma glucose value of greater than or equal to 11.1 mmol/L at 2 hours after the 75g glucose to lerance test; (3) Being conscious and capable of communicating and exchanging information with the researcher, (4) Voluntarily participating in this study. The exclusion criteria were as follows: (1) Combined with other serious acute diseases, requiring strict bed rest; (2) Presence of cognitive impairment, auditory dysfunction, etc. affecting the completion of the questionnaire; (3) Confirmed diagnosis of mental disorder.

According to the sample size estimation method of Kendall’s multi-factor study, the sample size should be 5 to 10 times the number of the studied variables. This study includes the type D personality scale (14 items), the social support scale (12 items), and the self-rating depression scale (20 items), totaling 46 factors. In this study, we took 10 times the number of the studied factors. Considering that there might be invalid questionnaires in the survey research, we increased the original sample size (460 people) by 10%. Therefore, the sample size to be surveyed should be at least 506 people. A total of 1,000 questionnaires were distributed in this study, with 936 valid questionnaires and a response rate of 93.6%.

### 2.2 Research tools

#### 2.2.1 Demographic characteristics

Demographic data includes gender, age, ethnicity, height, weight, education level, marital status, work situation, area of residence, family income, chronic disease, family history of diabetes, duration of diabetes, smoking, drinking, daily exercise time, daily sleep time, and types of diabetes complications.

#### 2.2.2 Type D personality scale

We used the type D personality scale developed by the Dutch psychologist Denollet (17). It consists of 14 items and 2 dimensions: negative emotions(seven items) and social inhibition(seven items). It is scored on a 5-point likert scale, ranging from “0-4” for “very non-conforming” to “very conforming.” Negative emotions and social inhibition with a total score of >10 are considered to be Type D personality. Cronbach’s α coefficient was 0.917.

#### 2.2.3 Perceived social support scale

The scale was developed by ZIMET et al (18). It has 12 entries and 3 dimensions: friend support, family support, and other support. The scale adopts a 7-point likert scale, with “1-7” indicating “strongly disagree” to “strongly agree”, and the total score is the sum of each item, and the higher the total score is, the higher the level of perceived social support is. The higher the total score, the higher the level of social support, of which, 61–84 is considered high comprehension social support, 37–60 is regarded as medium comprehension social support, and 12–36 is regarded as low comprehension social support. Cronbach’s α coefficient was 0.837.

#### 2.2.4 Self-rating depression scale

The scale developed by Zung in 1965 (19). The scale consists of 20 items and four dimensions: psycho-emotional symptoms, physical symptoms, psychomotor symptoms, and psychological symptoms, and adopts a 4-point likert scale ranging from 1 = ”rarely or none of the time” to 4 = ”most or all of the time”. The standardized score is less than 53 as no depression, 53–62 as mild depression, 63–72 as moderate depression, and more than 72 as severe depression. Cronbach’s α coefficient was 0.846.

### 2.3 Statistical analysis

Using SPSS 26.0, frequencies and constituent ratios were used for description, and the chi-square test was employed for statistical analysis. Variables with statistical significance (*P* < 0.05) were included in the logistic regression model and decision tree model to analyze how these variables affect depression in patients with diabetes. The logistic regression model used whether depression as the dependent variable, and the meaningful variables from the univariate analysis as the independent variables. The decision tree model used the CHAID algorithm, with a maximum tree depth of 3, a minimum sample size of 100 for parent nodes, and a minimum sample size of 50 for child nodes. The receiver operating characteristic (ROC) curves of the logistic regression model and decision tree model were drawn, and the differences between the two models were analyzed by comparing the area under the ROC curve (AUC), sensitivity, and specificity. Pearson correlation analysis was used to explore the relationship between Type D personality, social support, and depression in patients with diabetes. AMOS 24.0 was used to construct a structural equation model, with Type D personality as the independent variable, depression as the dependent variable, and social support as the mediating variable, and parameter estimation was performed using the maximum likelihood method.

### 2.4 Ethical declaration

This research complies with ethical standards and has been approved by the Ethics Committee of Shenyang Medical College.

## 3 Results

### 3.1 Univariate analysis of depression in patients with diabetes

The results of the *χ^2^* test analysis showed that there were no statistically significant differences in depression scores among ethnic group, educational level, average monthly family income per person, hypertension, hyperlipidemia, daily sleep time, family history of diabetes, insulin injection, and types of complications (*P* > 0.05); while gender, age, BMI, marital status, work situation, family location, coronary heart disease, stroke, smoking, passive smoking, drinking, daily exercise time, duration of diabetes, and oral hypoglycemic drugs did have statistically significant differences in depression scores (*P* < 0.01, *P* < 0.05). As shown in **Table 1**.

**Table 1:** Univariate analysis of depression in patients with type 2 diabetes.

Variates	Classification	Number	No depression	Depression	χ^2^	P
Gender	Male	503(53.70%)	464(55.00%)	87(41.90%)	5.787	<0.05
	Female	433(46.30%)	379(45.00%)	54(58.10%)		
Age	<60	400(42.70%)	376(44.60%)	24(25.80%)	12.092	<0.01
	≥60	536(57.30%)	467(55.40%)	69(74.20%)		
Ethnicity	Han Chinese	850(90.80%)	763(90.50%)	87(93.50%)	0.927	0.336
	Others	86(9.20%)	80(9.50%)	6(6.50%)		
BMI	<28.0	803(85.80%)	732(86.80%)	71(76.30%)	7.559	<0.01
	≥28.0	133(14.20%)	111(13.20%)	22(23.70%)		
Literacy level	High school and below	502(53.60%)	446(52.90%)	56(60.20%)	1.799	0.180
	Above high school	434(46.40%)	397(47.10%)	37(39.80%)		
Marital status	Married	827(88.40%)	738(87.50%)	89(95.70%)	5.413	<0.05
	Others	109(11.60%)	105(12.50%)	4(4.30%)		
Work situation	Retirement	627(67.00%)	556(66.00%)	71(76.30%)	4.088	<0.05
	Others	309(33.00%)	287(34.00%)	22(23.70%)		
Home location	Urban	674(72.00%)	619(73.40%)	55(59.10%)	8.484	<0.05
	Rural	262(28.00%)	224(26.60%)	38(40.90%)		
Monthly income	≤4000	605(64.60%)	542(64.30%)	63(67.70%)	0.436	0.509
	>4000	331(35.40%)	301(35.70%)	30(32.30%)		
Hypertension	Yes	611(65.00%)	542(64.30%)	69(74.20%)	3.621	0.057
	No	325(34.70%)	301(35.70%)	24(25.80%)		
Hyperlipidemia	Yes	94(10.00%)	86(10.20%)	8(8.60%)	0.237	0.626
	No	842(90.00%)	757(89.80%)	85(91.40%)		
Coronary heart disease	Yes	291(31.10%)	246(29.20%)	45(48.40%)	14.421	<0.01
	No	645(68.90%)	597(70.80%)	48(51.60%)		
Stroke	Yes	225(24.00%)	184(21.80%)	41(44.10%)	22.728	<0.01
	No	711(76.00%)	659(78.20%)	52(55.90%)		
Smoking	Yes	512(54.70%)	478(56.70%)	34(36.60%)	13.715	<0.01
	No	424(45.30%)	365(43.30%)	59(63.40%)		
Passive smoking	Yes	393(42.00%)	344(40.80%)	49(52.70%)	4.854	<0.05
	No	543(58.00%)	499(59.20%)	44(47.30%)		
Drinking alcohol	Yes	452(48.30%)	426(50.50%)	26(28.00%)	17.097	<0.01
	No	484(51.70%)	417(49.50%)	67(72.00%)		
Sleeping time	<8 hours	387(41.30%)	347(41.20%)	40(43.00%)	0.118	0.731
	≥8 hours	549(58.70%)	496(58.80%)	53(57.00%)		
Physical activity time	≤1hours	764(81.60%)	672(79.70%)	92(98.90%)	20.606	<0.05
	>1hours	172(18.40%)	171(20.30%)	1(1.10%)		
Family history of diabetes	Yes	535(57.20%)	482(57.20%)	53(57.00%)	0.001	0.972
	No	401(42.80%)	361(42.80%)	40(43.00%)		
Duration of diabetes	<10years	596(63.70%)	523(62.00%)	73(78.50%)	9.804	<0.01
	≥10years	340(36.30%)	320(38.00%)	20(21.50%)		
Oral hypoglycemic drugs	Yes	443(47.30%)	408(48.40%)	35(37.60%)	3.893	<0.05
	No	493(52.70%)	435(51.60%)	58(62.40%)		
Insulin injection	Yes	486(51.90%)	434(51.50%)	52(55.90%)	0.659	0.417
	No	450(48.10%)	409(48.50%)	41(44.10%)		
Diabetes complications	≤2 kinds	636(67.90%)	567(67.30%)	69(74.20%)	1.849	0.174
	>2 kinds	300(32.10%)	276(32.70%)	24(25.80%)		

*p<0.05, **p<0.01.

### 3.2 Binary logistic regression analysis of the influencing factors of depression in patients with diabetes

Taking whether patients with diabetes are depressed as the dependent variable (0 = no, 1 = yes), and using 14 independent variables including gender, age, BMI, marital status, work situation, family location, coronary heart disease, stroke, smoking, passive smoking, drinking, daily exercise time, duration of diabetes, and oral hypoglycemic drugs as independent variables, a binary logistic regression analysis was conducted. The results showed that gender, age, marital status, family location, coronary heart disease, stroke, and exercise time were the main factors contributing to depression in patients with diabetes. Among them, female gender, age ≥ 60 years old, rural location, having coronary heart disease, having stroke, and daily exercise time ≤ 1 hour were risk factors for depression in patients with diabetes, while other marital statuses were protective factors for depression. As shown in **Table 2**.

**Table 2:** Binary logistic regression analysis of factors affecting depression in diabetic patients.

Variates	Classification	B	SE	*Wald*	*P*	OR	95%CI	
Gender	Male vs Female	0.605	0.248	5.977	P<0.05	1.832	1.128	2.976
Age	<60 vs ≥60	1.648	0.534	9.534	P<0.01	5.198	1.826	14.8
BMI	<28.0 vs ≥28.0	0.452	0.293	2.378	0.123	1.572	0.885	2.792
Marital status	Married vs Others	-1.363	0.552	6.092	P<0.05	0.256	0.087	0.755
Work situation	Retirement vs Others	0.803	0.542	2.197	0.138	2.233	0.772	6.459
Home location	Urban vs Rural	0.541	0.247	4.814	P<0.05	1.718	1.059	2.785
Coronary heart disease	No vs Yes	0.721	0.25	8.306	P<0.01	2.057	1.260	3.359
Stroke	No vs Yes	0.875	0.246	12.635	P<0.01	2.398	1.481	3.885
Smoking	No vs Yes	-0.009	0.334	0.001	0.977	0.991	0.514	1.908
Passive smoking	No vs Yes	0.229	0.254	0.815	0.367	1.258	0.764	2.069
Drinking alcohol	No vs Yes	-0.641	0.373	2.955	0.086	0.527	0.254	1.094
Physical activity time	>1hours vs ≤1hours	2.623	1.025	6.543	P<0.05	13.77	1.846	102.718
Duration of diabetes	≥10years vs <10years	-0.022	0.303	0.005	0.943	0.979	0.54	1.773
Oral hypoglycemic drugs	No vs Yes	-0.279	0.249	1.254	0.263	0.757	0.465	1.232

### 3.3 Decision tree model for influencing factors of depression in patients with diabetes

**Figure 1:**
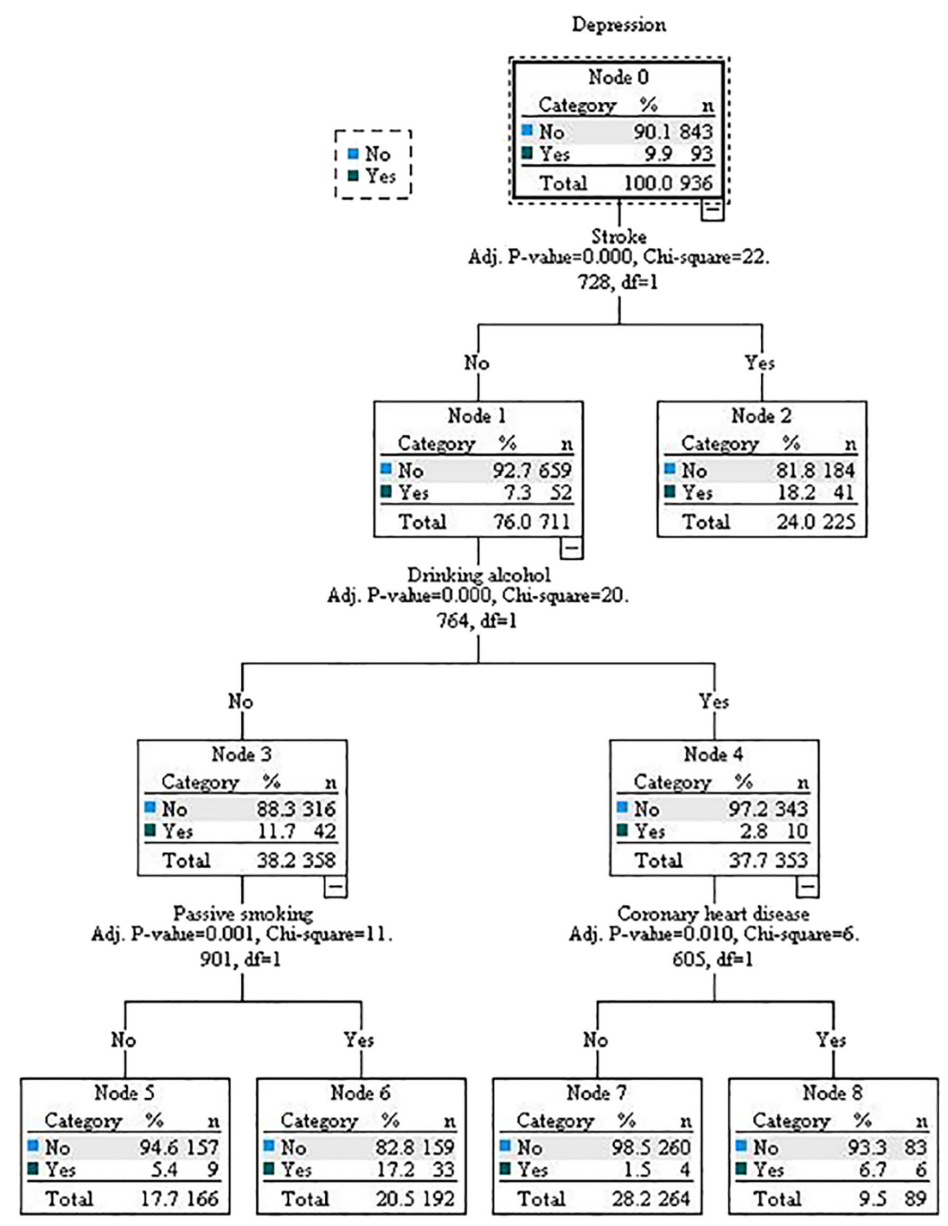
Decision tree model.

The decision tree model for the influencing factors of depression in patients with diabetes was grown into 4 layers, with 9 nodes and 5 terminal nodes, as shown in **Figure 1**. The model results indicated that stroke, alcohol consumption, passive smoking, and coronary heart disease were important influencing factors for depression. Among them, the root node was age, indicating that the correlation between stroke and depression was the highest. Among patients with diabetes, the depression detection rate was higher in those with a history of stroke (18.2%) than in those without (7.3%). The second layer variable of the decision tree was alcohol consumption. In the subgroup without stroke, the depression detection rate of patients with diabetes who did not drink alcohol was higher (11.70%). The third layer variable was passive smoking and coronary heart disease. Among patients with diabetes who did not consume alcohol, the depression detection rate was higher in those exposed to passive smoking (17.2%) than in those not exposed (5.4%). Among those who consumed alcohol, the rate was higher in patients with diabetes with coronary heart disease (6.7%) than in those without (1.5%).

### 3.4 Comparison of logistic regression model and decision tree model for factors affecting depression in patients with diabetes

**Figure 2:**
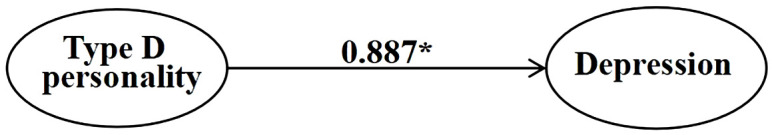
Direct effect of type D personality and depression in diabetics.

Both the logistic regression and decision tree model results indicated that stroke and coronary heart disease were the main influencing factors for depression in patients with diabetes. By using the predicted probabilities obtained from the two models as the test variables to draw the ROC curve, as shown in **Figure 2**, it can be seen that the effects of the two models are similar, but there are also certain differences. Gender, age, marital status, place of residence, and daily exercise time were statistically significant in the logistic regression analysis, but were excluded in the decision tree model. In the ROC curve, the area under the curve of the logistic regression analysis was 0.811 (95% CI: 0.722, 0.849), with a sensitivity of 79.60% and a specificity of 72.00%; the area under the curve of the decision tree model was 0.717 (95% CI: 0.671, 0.764), with a sensitivity of 79.60% and a specificity of 59.30%, both of which were statistically significant (*P* < 0.05), as shown in **Table 3**. The area under the curve of the logistic regression analysis was slightly larger than that of the decision tree model, indicating that the effect of logistic regression analysis was better than that of the decision tree.

**Table 3:** ROC curve indicators of the logistic regression model and the decision tree model.

Classification of model	AUC	95%CI	Cut-off	Sensitivity(%)	Specificity(%)
Logistic regression model	0.811	0.772,0.849	0.516	79.60	72.00
Decision tree	0.717	0.671,0.764	0.483	79.60	59.30

### 3.5 Correlation among study variables

Pearson correlation analysis of type D personality, social support, and depression in patients with diabetes showed that type D personality was significantly positively correlated with depression (*r* = 0.464, *P* < 0.01), type D personality was significantly negatively correlated with social support (*r* = -0.356, *P* < 0.01), and social support was significantly negatively correlated with depression (*r* = -0.322, *P* < 0.01). As shown in **Table 4**.

**Table 4:** Correlation analysis of type D personality, social support, and depression.

Variables	Type D personality	Social support	Depression
Type D personality	1		
Social support	-0.356**	1	
Depression	0.464**	-0.322**	1

**p<0.01.

### 3.6 Structural equation modeling analysis of type D personality, social support, and depression

A structural equation model was constructed using AMOS 24.0, taking type D personality in patients with diabetes as the independent variable, social support as the mediator, and depression as the dependent variable. The results of the model showed that type D personality not only directly affects depression, but also has a significant indirect effect on depression through social support. The direct path of type D personality affecting depression is shown in **Figure 2**, indicating a significant effect of type D personality on depression (*β* = 0.887, *P* < 0.01). All the fit indices of the model were met(χ^2^/df=3.514<5,CFI= 0.914>0.9, RMSEA = 0.064<0.10, TLI = 0.905>0.9, IFI = 0.917>0.9, NFI = 0.894>0.8).

**Figure 3:**
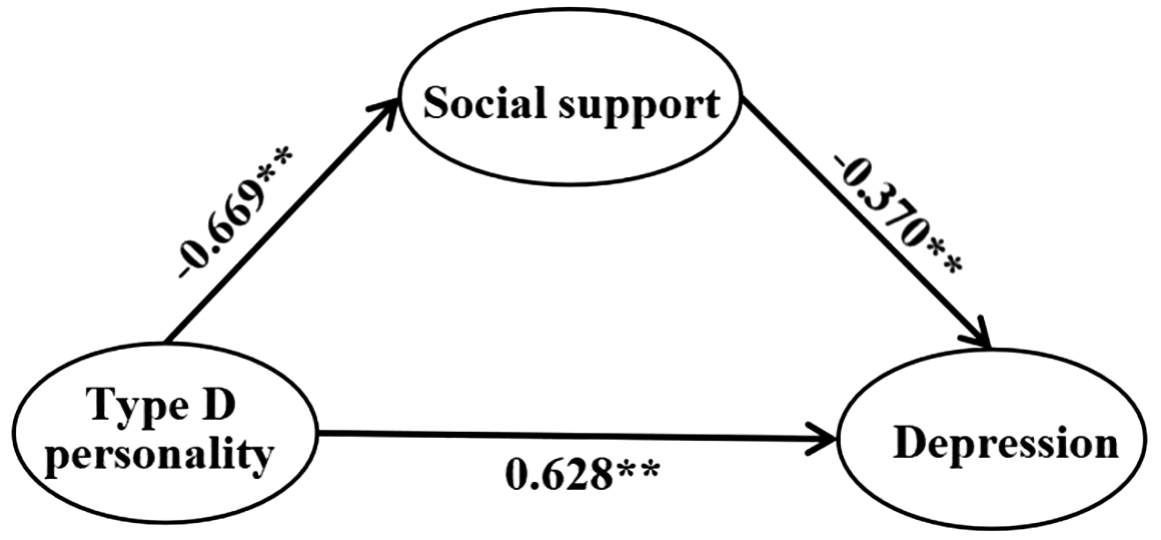
Structural equation modeling of social support in type D personality and depression in diabetics.

The indirect pathway from Type D personality to depression mediated by social support is shown in **Figure 3**. All the fit indices of the model were met(χ^2^/df=4.853<5,CFI= 0.879>0.8, RMSEA = 0.064<0.10, TLI = 0.870>0.8, IFI = 0.880>0.8, NFI = 0.853>0.8). The results showed that social support was significantly associated with both type D personality (*β* = -0.669, *P* < 0.01) and depression (*β* = -0.370, *P* < 0.01). The absolute value of the path coefficient between type D personality and depression decreased when social support was used as a mediator (*β* = 0.628, *P* < 0.01), and in the present study, controlling for the mediator variable, the effect of the independent variable on the dependent variable decreased, suggesting that social support plays a mediating role in the relationship between type D personality and depression.

From the results of the SEM model path test, it can be seen that there is a negative correlation between type D personality and social support in patients with diabetes, and there is a positive correlation with depression; there is a negative correlation between social support and depression. The path coefficients and their specific results are shown in **Table 5**.

**Table 5:** Structural equation modeling path relationship test results.

Path	Estimate	S.E.	C.R.	P
Type D personality→Social support	-0.669	0.073	-14.179	<0.01
Type D personality→Depression	0.628	0.033	3.710	<0.01
Social support→Depression	-0.370	0.016	-2.932	<0.01

## 4 Discussion

### 4.1 Psychological and social risk factors and underlying mechanisms associated with the development of diabetes in individuals with type D personality

The type D personality, as a stable psychological trait, is characterized by negative emotional tendencies and social inhibition. In patients with diabetes, this trait significantly increases the risk of depression and complicates disease management through interconnected psychosocial and biological mechanisms. At the behavioral level, persistent negative emotions and social inhibition impair treatment adherence and self-management capacity, manifesting as inconsistent medication use, irregular blood glucose monitoring, poor dietary control, and reduced engagement in social interactions and group activities—ultimately leading to decreased daily physical activity. At the physiological level, individuals with type D personality and diabetes often experience chronic stress, resulting in sustained activation of the hypothalamic-pituitary-adrenal axis and excessive cortisol secretion. Elevated cortisol levels not only reduce insulin sensitivity and promote hepatic gluconeogenesis and glycogenolysis, thereby exacerbating glycemic fluctuations, but also suppress immune function and increase susceptibility to complications. Furthermore, negative emotions and diminished self-efficacy contribute to a vicious cycle that reinforces negative perceptions of health status, intensifying emotional distress and psychological burden. Consequently, through dual pathways—behavioral maladaptation and neuroendocrine dysregulation—type D personality contributes to poor glycemic control, higher complication rates, and increased vulnerability to depression, illustrating the complex interplay between psychological traits and metabolic dysfunction within a multifactorial framework.

### 4.1 Higher prevalence of depression in patients with diabetes

The results of this study showed that the prevalence of depressive symptoms in patients with diabetes was 49.1%, consistent with previous findings (20). This rate was notably higher than the 12.3% reported in an international study by Angela Chieh (21) and the 21.3% reported for hospitalized patients with type 2 diabetes in another study (22). This difference may be because the present study used a larger sample size to reduce sampling error and improve the representativeness and accuracy of the findings, thus revealing a more complete picture of the prevalence and severity of depression in the patients with diabetes population. There are differences in the prevalence of depression, which may also be related to different regions, different populations, changes in medical conditions depression screening methods, so it is extremely important to explore the influencing factors of depression in patients with diabetes.

This study employed the Self-Rating Depression Scale, which is widely used among various populations and is applicable to diabetic inpatients. This scale has been used in previous studies to assess the depressive symptoms of diabetic patients and has demonstrated good reliability and validity (23). In this study, the Cronbach’s α coefficient of SDS was 0.917, indicating that it has good internal consistency within the sample and supports its reliability for use in hospitalized diabetic patients. Hospitalized patients often have more severe disease conditions, acute complications, or are undergoing treatment, which may contribute to higher levels of psychological stress compared to community-dwelling diabetic patients, leading to an increased prevalence of detected depression. Furthermore, the hospital environment may restrict patients’ daily activities and social interactions, potentially impairing their perceived social support and emotional well-being. Future research should systematically compare depression levels between hospitalized and non-hospitalized patients with diabetes to clarify the influence of clinical setting on mental health outcomes.

### 4.2 Factors influencing depression in patients with diabetes

By combining the analysis results of the logistic regression and decision tree models, it was found that stroke and coronary heart disease are the main factors contributing to depression. Gender, age, marital status, place of residence in the family, and daily exercise time have statistical significance in the logistic regression model, but they did not enter the decision tree model. On one hand, this might be due to the depth of the decision tree and the sample size limit of each node. Some potential influencing factors failed to be fully manifested in the current model and might be captured in a deeper decision tree model. On the other hand, it could be that the influence of some variables is relatively small and they were eliminated during the model growth and pruning process. However, existing studies have confirmed that gender, age, place of residence in the family, and exercise time are closely related to depression (24–26). Patients with diabetes suffering from coronary heart disease and stroke are more prone to depression, mainly due to the severity and suddenness of cardiovascular and cerebrovascular diseases, which leads to a decrease in the quality of life of patients with diabetes, increasing their tension and psychological burden, which in turn leads to depressive symptoms (27). In this study, the efficacy of the logistic regression model was superior to that of the decision tree model. However, the sensitivities of the two models were the same. In practical applications, these two models can complement each other and provide valuable references and guidance for the mental health work of undergraduate students majoring in medical and elderly care health from different perspectives. In this study, the efficacy of the logistic regression model was superior to that of the decision tree model. However, the sensitivities of the two models were the same. In practical applications, these two models can complement each other and provide valuable references and guidance for the mental health work of undergraduate students majoring in medicine and geriatric care from different perspectives. The results of the logistic regression model offer precise influence coefficients, which help to deeply understand the direction and degree of each factor’s impact on depression. The decision tree model provides an intuitive decision process and a clear tree structure, capable of identifying key decision paths and variable combinations. The combination of these two models can improve the reliability and robustness of the analysis results by comparing the consistency of different prediction results, providing a novel method for a more comprehensive study of the impact of depression in diabetic patients and the predictive factors.

### 4.3 Relationship between type D personality and depression in patients with diabetes

The results of Pearson’s correlation analysis in this study showed a significant positive correlation between type D personality and depression in patients with diabetes (*P* < 0.01), the higher the score of type D personality, the more severe the patients’ depression, which is consistent with the results of a cross-sectional study (28). The results of regression and structural equation modeling showed that type D personality in Chinese patients with diabetes has a direct effect on the occurrence of depression, which can directly lead to an increased risk of depression. This finding is consistent with the results of a prospective observational study by Yamaguchi et al., which enrolled 89 patients with coronary heart disease. Using logistic regression analysis, the study found a significant positive association between Type D personality and depressive mood (23). Type D personality is mainly manifested in two aspects, namely, negative emotions and social inhibition, and related studies have shown that people with high levels of negative emotions and social inhibition are more likely to experience worry, anger, frustration, and despair (29) patients with diabetes with type D personality are interested in the disease itself, excessive worry about possible complications and treatment costs may exacerbate their negative emotions such as anxiety and pessimism, which in turn may lead to depressive symptoms. At the same time, long-term blood glucose monitoring, medication, and lifestyle management may cause patients with diabetes to feel depressed, tired, and frustrated. Patients with type D personality have difficulties in socialization, which may lead to loneliness and exclusion in their interactions with others, further exacerbating the depressive mood.

Type D personality is a significant psychosocial risk factor, particularly among patients with cardiovascular diseases, and is strongly associated with adverse prognosis and diminished psychological well-being. This personality trait not only directly impairs emotion regulation but may also indirectly increase the likelihood of depression onset. Characterized by negative emotional tendencies and social inhibition, individuals with type D personality are more vulnerable to chronic stress, which in turn elevates susceptibility to negative emotional states such as depression. The progression from a stable personality disposition to recurrent emotional distress influences how individuals perceive and respond to stress, often resulting in avoidance or passive coping strategies that further intensify psychological strain. Furthermore, psychosocial risk factors rarely occur in isolation; instead, they tend to co-occur within individuals, creating an interplay among personality, emotional functioning, and stress responses. This dynamic interaction collectively impacts mental health outcomes, contributing to the emergence and persistence of depressive symptoms. Therefore, attention should be paid to the type D personality tendency in patients with diabetes to avoid the impact of type D personality on their mental health.

### 4.4 Relationship between social support and depression in patients with diabetes

The main effect model in the social support theory believes that social support can promote the physical and mental health of individuals, the level of social support felt by individuals is positively correlated with the level of physical and mental health, and the results of Pearson’s correlation analysis in the present study showed that the social support of patients with diabetes was significantly negatively correlated with depression (*P* < 0.01), patients with a stronger ability to comprehend social support had a lower degree of depression, which is consistent with the theory. The results of regression and structural equation modeling showed that social support in Chinese patients with diabetes had a direct effect on the occurrence of depression, which could directly reduce the risk of depression and was a protective factor for depression, which was consistent with the results of previous studies (30, 31). Social support is associated with the development of diabetes-related problems and psychological well-being, and when patients with diabetes comprehend social support, they feel cared for, accepted, and appreciated, their subjective well-being rises, and they adopt a positive attitude toward their chronic disease (32). The provision of social support by spouses, family, and friends may influence the attitude of patients with diabetes towards the disease, and patients with greater ability to comprehend social support are more relaxed in their psychological state and deal with their health problems more positively.

### 4.5 The mediating role of social support between type D personality and depression

The buffering effect model of social support theory suggests that individuals who receive psychological or material support in social relationships can mitigate the negative effects of other factors on physical and mental health, and the findings of this study are consistent with this theory, type D personality not only has a direct effect on depression in patients with diabetes but also has an indirect effect on depression through the mediating path of social support. The present study showed that patients with diabetes with D personality were more likely to experience depressive symptoms, and improving their ability to feel social support may reduce the probability of depressive symptoms, which is consistent with a study in China (33). Social support is a mediator between type D personality and depressive mood, probably because patients with type D personality traits have the manifestation of social inhibition, which suppresses the expression of emotions and behaviors in social interaction, leading to higher levels of social detachment in the long term, which in turn may lead to a decrease in the social support they receive and a decrease in the utilization of social support, resulting in negative moods such as anxiety and depression (34). Therefore, attention should be paid to the social support of patients with diabetes with type D personality traits, which can be used to promote the physical and mental health of patients with diabetes by providing more social support with the improvement of the patient’s ability to comprehend social support.

First, type D personality has been reported as a risk factor for depression in patients with cardiovascular and cerebrovascular diseases. However, this study, through a structural equation model, clearly confirmed that in the group of hospitalized diabetic patients, type D personality has a positive effect on depressive mood. Second, through a large sample survey and regression analysis, this study further confirmed the negative effect of social support on depression in diabetic patients. Third, it deeply explored the intrinsic relationship between type D personality and social support in diabetic patients. The research results show that diabetic patients with type D personality have significantly lower levels of perceived social support. That is, type D personality not only directly causes emotional distress but may also indirectly affect their social support system. Fourth, an intermediary model was constructed and verified in diabetic patients, indicating that social support plays a partial mediating role between type D personality and depression. That is, type D personality can directly increase the risk of depression and also indirectly cause depression by reducing patients’ ability to perceive social support.

## 5 Limitation

At present, there are limitations in this present study. First, only one city was selected for this study, which may limit the generalizability of the study. Second, the results of this study may be limited by unmeasured confounders such as individual social status and regional differences in health levels.

## 6 Conclusion

This study, focusing on hospitalized patients with diabetes in China, employed structural equation modeling to confirm that social support partially mediates the relationship between Type D personality and depression. These findings indicate that Type D personality not only directly increases the risk of depression but also indirectly contributes to depression by diminishing patients’ perceived levels of social support. Moreover, the study revealed that the prevalence of depressive symptoms among hospitalized diabetic patients in China is substantially higher than that observed in most outpatient or community-based populations, underscoring the severity of mental health issues within this clinical group. Furthermore, by comparing logistic regression and decision tree models, the study demonstrated that logistic regression offers superior predictive accuracy for this type of data, thereby providing a methodological reference for future research.

## Data Availability

The raw data supporting the conclusions of this article will be made available by the authors, without undue reservation.
